# Connexin 43 plays a role in proliferation and migration of pulmonary arterial fibroblasts in response to hypoxia

**DOI:** 10.1177/2045894020937134

**Published:** 2020-07-06

**Authors:** Andrew J. McNair, Kathryn S. Wilson, Patricia E. Martin, David J. Welsh, Yvonne Dempsie

**Affiliations:** 1 Department of Biological and Biomedical Science, School of Health and Life Sciences, Glasgow Caledonian University, Glasgow, UK; 2 Institute of Cardiovascular and Medical Sciences, College of Medical, Veterinary and Life Sciences, University of Glasgow, Glasgow, UK

**Keywords:** pulmonary hypertension, fibroblast, hypoxia, connexins

## Abstract

Pulmonary hypertension (PH) is a disease associated with vasoconstriction and remodelling of the pulmonary vasculature. Pulmonary artery fibroblasts (PAFs) play an important role in hypoxic-induced remodelling. Connexin 43 (Cx43) is involved in cellular communication and regulation of the pulmonary vasculature. Using both *in vitro* and *in vivo* models of PH, the aims of this study were to (i) investigate the role of Cx43 in hypoxic-induced proliferation and migration of rat PAFs (rPAFs) and rat pulmonary artery smooth muscle cells (rPASMCs) and (ii) determine whether Cx43 expression is dysregulated in the rat sugen5416/hypoxic model of PH. The role of Cx43 in hypoxic-induced proliferation and migration was investigated using Gap27 (a pharmacological inhibitor of Cx43) or genetic knockdown of Cx43 using siRNA. Cx43 protein expression was increased by hypoxia in rPAFs but not rPASMCs. Hypoxic exposure, in the presence of serum, resulted in an increase in proliferation of rPAFs but not rPASMCs. Hypoxic exposure caused migration of rPAFs but not rPASMCs. Phosphorylation of p38 mitogen-activated protein kinase (MAPK) and ERK1/2 were increased by hypoxia in rPAFs. The effects of hypoxia on proliferation, migration and MAPK phosphorylation in rPAFs were attenuated in the presence of Gap27 or Cx43 siRNA. Cx43 protein expression was increased in sugen5416/hypoxic rat lung; this increased expression was not observed in sugen5416/hypoxic rats treated with the MAPK pathway inhibitor GS-444217. In conclusion, Cx43 is involved in the proliferation and migration of rPAFs in response to hypoxia via the MAPK signalling pathway.

Pulmonary hypertension (PH) is a complex vascular disease which is characterised by an increase in pulmonary arterial pressure (PAP) and remodelling of the pulmonary arteries.^1^ PH develops in response to hypoxia in most species including humans,^2^ and as such hypoxia has been a commonly used model of PH for decades, both *in vitro* and *in vivo*. Exposure to hypoxia results in an acute increase in PAP due to hypoxic pulmonary vasoconstriction. This is followed by pulmonary vascular remodelling leading to sustained increases in PAP.^3^ The pulmonary artery is composed of three main cell types: fibroblasts, smooth muscle and endothelial cells. Pulmonary artery fibroblasts (PAFs) play a major role in pulmonary vascular remodelling, particularly in response to hypoxia. They have been shown to proliferate and migrate in response to hypoxia and mediate the proliferation and migration of pulmonary artery smooth muscle cells (PASMCs).^2^

Connexins and pannexins are transmembrane proteins known to play an important role in cellular communication. Oligomerisation of these proteins leads to the formation of membrane-associated channels that can mediate the release of small chemical signalling molecules such as adenosine triphosphate, cyclic adenosine monophosphate and cyclic guanosine monophosphate or ions such as calcium from the cell cytosol to the extracellular milieu.^4^ Connexin channels from neighbouring cells can align to form a gap junction allowing transfer of small molecules directly from the cytoplasm of one cell to that of another.^5^ Connexins 37 (Cx37), 40 (Cx40), 43 (Cx43), 45 (Cx45) and pannexin-1 (Panx1) are expressed throughout the vascular system, and their role in regulation of the systemic vasculature is well documented.^6^ Interestingly, recent evidence suggests a role for connexins in regulation of the pulmonary vasculature. Cx37 and Cx40 expression are downregulated in pulmonary artery endothelial cells (PAECs) from pulmonary arterial hypertension (PAH) patients.^7^ In addition, mice genetically deficient in Cx40 are protected against hypoxic-induced PH and hypoxic pulmonary vasoconstriction.^8^ Cx43 levels are increased in pulmonary arteries from patients with PH associated with hypoxemic chronic lung disease, while Cx43 levels are decreased in pulmonary arteries from patients with idiopathic PAH. In addition, mice genetically deficient in Cx43 are partially protected against hypoxic-induced PH.^9^ Pharmacological inhibition of Cx43 can reduce phenylephrine-induced contraction of pulmonary arteries derived from both chronic hypoxic and monocrotaline rats and also reduce 5-hydroxytryptamine (5-HT)-induced contraction of pulmonary arteries derived from control rats.^10^ Mice deficient in Cx43 show reduced pulmonary vascular relaxation, and pharmacological inhibition of Cx43 also reduces pulmonary vascular relaxation.^11^ In addition, Cx43 can mediate 5-HT signalling between rat PAECs and rat PASMCs (rPASMCs).^12^ Transfer of 5-HT between these cell types was inhibited by the non-specific gap junction blocker, carbenoxolone or by siRNA knockdown of Cx43, but not by the serotonin transporter inhibitor fluoxetine.^13^ The nitric oxide synthase inhibitor, asymmetric dimethyl arginine (ADMA), is upregulated in PAH patients. ADMA has been shown to inhibit protein expression and membrane localisation of Cx43 in pulmonary endothelial cells. The effect of ADMA on reduced gap junctional communication is prevented by over-expression of Cx43 or by treatment with rotigaptide, which enhances connexin coupling.^14^

In HeLa cells transfected to express Cx43, and mouse systemic vascular smooth muscle cells, Cx43 mediated proliferation and migration via downstream activation of p38 mitogen-activated protein kinase (MAPK).^15,16^ Previous work from our laboratory has determined that activation of this pathway plays a crucial role in PAF proliferation in response to acute hypoxia. For example, phosphorylation of p38 MAPK is upregulated in PAFs in response to hypoxia, and inhibition of p38 MAPK attenuates hypoxic-induced proliferation and migration of PAFs.^2^ It has also been shown that inhibition of the MAPK pathway leads to decreased proliferation, migration and p38 MAPK expression in rat PAFs (rPAFs) and also attenuates the development of PH in sugen5416/hypoxic rats.^17^ Apoptosis signal-regulating kinase-1 (ASK-1) is an upstream regulator of the MAPK pathway. In response to oxidative stress, ASK-1 undergoes auto-phosphorylation and activation of downstream p38 MAPK.^16^ Inhibition of ASK-1 leads to decreased proliferation, migration and p38 MAPK expression in rPAFs and also attenuates the development of PH in sugen5416/hypoxic rats.^16^

Until now, investigations of connexin expression and function in the pulmonary vasculature have focussed on PAECs and PASMCs. For example, Cx37, Cx40 and Cx43 are expressed in rat PAECs (rPAECs), while Cx37 and Cx43 are expressed in rPASMCs.^18^ Functional myo-endothelial gap junctions were demonstrated by using co-cultures of rPAECs and rPASMCs.^12^ However, to the best of our knowledge, the expression and function of connexins in PAFs have yet to be investigated. Thus, in this study, we cultured rPAFs and (for comparison) rPASMCs and examined gene and/or protein expression of Cx37, Cx40, Cx43, Cx45 and Panx1. We subsequently determined the effects of the connexin mimetic peptide Gap27, which is targeted to the SRPTEKTIFII sequence (amino acids 204–214) on the second extracellular loop of Cx43 and inhibits both Cx37 and Cx43, on hypoxic-induced proliferation, migration and activation of MAP kinase pathways in rPAFs.^19–21^ In addition to pharmacological inhibition with Gap27, we also investigated the effects of genetic knockdown of Cx43 using siRNA. As well as performing in vitro experiments using primary pulmonary vascular cells, we were also interested in determining if Cx43 expression was dysregulated in a rodent model of PH. We chose the sugen5416/hypoxic rat as this model capitulates the vascular lesions observed in human disease and is thought to be more robust than the hypoxic rodent model.^22^ Thus, we investigated Cx43 expression in whole lung tissue derived from sugen5416/hypoxic rats, including a subgroup dosed with an ASK-1 inhibitor.

## Methods

### Materials

All reagents were of Analar grade and were obtained from Sigma (Poole, Dorset, UK) unless specified otherwise. All tissue culture flasks and media were obtained from Invitrogen (Paisley, Renfrewshire, UK). Foetal bovine serum (FBS) was obtained from Imperial Laboratories (Andover, Hants, UK). Rabbit polyclonal antibodies specific for the activated dual phosphorylated forms of the two MAP kinase family members (ERK1/ERK2) (Thr202/Tyr204) and p38 MAPK (Thr180/Tyr182) and their appropriate control antibodies were obtained from Cell Signalling Technology (Danvers, MA, USA). Rabbit polyclonal antibodies for Cx37, Cx40 and Cx43 were obtained from Alpha Diagnostics (San Antonio, TX, USA), Invitrogen (Paisley, Renfrewshire, UK) and Sigma (Poole, Dorset, UK), respectively. Secondary goat anti-rabbit horseradish peroxidase (HRP) antibody was obtained from New England Biolabs (Ipswich, MA, USA). Gap27 was purchased from AdooQ (Irvine, CA, USA) and the Cx43 siRNA from Integrated DNA Technologies (Skokie, IL, USA).

### Animals

All animal procedures were carried out in accordance with Directive 2010/63/EU of the European Parliament. Ethical approval was granted from the University of Glasgow Animal Welfare and Ethical Review Board (P965255C4).

Primary rPAFs and rPASMCs were obtained from adult male Sprague-Dawley rats (3 months of age) maintained in normoxic conditions on a 12 h light/dark cycle and allowed free access to standard diet and water. Rats were euthanised by exsanguination under terminal anaesthesia (5% isoflurane supplemented in O_2_) and heart and lung tissue dissected free.

### Sugen5416/hypoxic rat model of PH

Male Sprague-Dawley rats (4 weeks old) received a subcutaneous injection of sugenSU5416 (20 mg/kg) prior to 2 weeks of hypobaric hypoxia (550 mbar) and a subsequent 3-week normoxic phase. Control rats were dosed with a vehicle substance (0.5% (w/v) carboxymethylcellulose sodium, 0.9% (w/v) sodium chloride, 0.4% (v/v) polysorbate 80, 0.9% (v/v) benzyl alcohol in deionised water). One group of rats received the ASK-1 inhibitor GS-444217 administered in the chow (a maximum of 10 µM) during the 3-week normoxic phase. The control groups received standard chow. Rats were euthanised at the end of the 3-week normoxic phase by anaesthetic overdose (5% isoflurane supplemented in O_2_). Heart and lung tissue were dissected free.

### Primary culture of rPAFs and rPASMCs

Main and branch pulmonary artery (diameter ∼0.5–1.5 mm) was dissected free from freshly excised rat lungs. The section was then cut longitudinally and opened into a flat sheet. The fibroblasts were isolated using a modified technique of Freshney.^23^ Muscular tissue and endothelial cell layers were removed by gentle abrasion of the vessel using a razor blade. The adventitia was dissected into approximately 1 mm^3^ portions and evenly distributed over the base of a 25 cm^2^ culture flask containing 2 ml of Dulbecco’s modification of Eagle’s medium (DMEM) supplemented with 20% FBS, with penicillin/streptomycin (400 iu/ml and 400 µg/ml) and L-glutamine (2 mM) until cellular outgrowth was observed. We have previously shown by staining for vimentin that this technique provides a pure culture of fibroblasts.^2^

rPASMCs were prepared using a similar technique; however, the intimal and adventitial layers were not removed by gentle abrasion as with the rPAF preparation. Staining for α smooth muscle actin proved a pure culture of smooth muscle cells.

rPAFs and rPASMCs were maintained in DMEM containing 10% FBS, supplemented with penicillin/streptomycin (200 iu/ml and 200 µg/ml) and L-glutamine (27 mg/ml) and used between Passage 3 and 7.

### Growth of cells in a hypoxic environment

A humidified temperature controlled incubator (Wolf Galaxy R, Nottingham, UK) was used as a hypoxic chamber. For hypoxic experiments, primary cultures of rPAFs or rPASMCs were quiesced in serum-free media (SFM) for 24 h then transferred to hypoxic conditions (5% O_2_, 5% CO_2_ and balance N_2_) over a 24-h experimental period. Over the 24-h experimental period, cells were incubated with SFM or media supplemented with 0.1% or 1% FBS. We have previously shown that rPAFs proliferate in response to hypoxia in the presence of 1% FBS but not 0.1% FBS.^22^

### Western blotting

rPAFs and rPASMCs were grown to 90% confluency in 6-well plates and quiesced for 24 h in SFM. The cells were then stimulated with 1% FBS and placed in either the normoxic or hypoxic incubator for 24 h. Reactions were terminated by the addition of 50-µl ice-cold radioimmunoprecipitation assay buffer (50 mM Tris (pH 7.4), 150 mM sodium chloride, 2% (v/v) NP 40, 0.25% (w/v) sodium deoxycholate, 1 mM EGTA (ethylene glycol-bis(β-aminoethyl ether)-N,N,N',N'-tetraacetic acid), 10 mM sodium orthovanadate, 0.5 mM phenylmethylsulfonyl fluoride, chymostatin (10 mg/ml), leupeptin (10 mg/ml), antipain (10 mg/ml) and pepstatin A (10 mg/ml)) and kept on ice for 30 min to facilitate the extraction of cellular proteins. The samples were centrifuged at 12,000 g for 15 min, and the resulting supernatants containing the solubilised proteins were used for Western blot analysis. Protein content of the samples was determined by the Micro BCA Protein Reagent Kit (Pierce, IL, USA). Samples (50 µg) were reduced and electrophoresed on 10% sodium dodecyl sulfate-polyacrylamide gel electrophoresis resolving gels under reducing conditions. The resolved proteins were transferred to polyvinylidene difluoride (Millipore, Watford, UK) and blocked at room temperature with 10% (w/v) non-fat dried milk in phosphate-buffered saline (PBS)/Tween 20 (v/v 0.1%) under constant agitation. Primary antibody was incubated with the blot for at least 1 h at room temperature. The blots were then washed in PBS/Tween before incubating with goat anti-rabbit IgG HRP in 5% (w/v) non-fat dried milk for 1 h with constant agitation. For each member of the MAPK family studied, activation was determined by reactivity with the antibody to the dually phosphorylated protein and compared to the control antibody which recognised both the active and inactive forms of the protein (total MAPK), and a ratio was calculated. The blots were thoroughly washed and then incubated with enhanced chemiluminescence (ECL) reagent (Amersham Life Sciences, UK) and exposed to film. Image J software was used to attribute densitometry values to quantify the results.

### RNA analysis

#### RNA extraction

rPAFs and rPASMCs were grown to 90% confluency in 6-well plates and quiesced for 24 h in SFM. Cells were then stimulated with 1% FBS and placed in either the normoxic or hypoxic incubator for 24 h. To extract RNA from rPAFs and rPASMCs, lysis was performed using QIAzol lysis reagent and extracted with the miRNEASY extraction kit according to manufacturer’s instructions. Initially, 700 μl QIAzol was added to the cells, and they were scraped as previously described. For RNA extraction, chloroform was added, and tubes were shaken vigorously for separation of RNA from DNA, proteins and lipids. The RNA was then precipitated with 100% RNA-free ethanol. The sample was transferred onto a spin column provided by the manufacturer to undergo spin column-based nucleic acid purification. Samples were analysed by a NanoDrop, ND-1000 spectrophotometer (Thermo Scientific, UK) to determine RNA concentrations.

#### Reverse transcription

Extracted RNA samples were reverse transcribed using TaqMan® reverse transcription reagents (Applied Biosystems, CA, USA) as per manufacturers’ instructions. [AW: RT has been expanded as Reverse Transcriptase buffer. Kindly confirm that this is correct or edit as needed.] Approximately 1 µg RNA was transcribed per sample in a cocktail of Reverse Transcriptase buffer, 25 mM magnesium chloride (MgCl_2_), deoxynucleotide triphosphates, random hexamers, RNase inhibitors and Multiscribe. Reverse transcription was performed using the Veriti® Thermal Cycler (Life Technologies, Paisley, UK) under the following cycling conditions: 10 min at 25℃ to maximise primer RNA template binding, 30 min at 48℃ for reverse transcription and 5 min at 95℃ to deactivate reverse transcription.

#### Quantitative real-time polymerase chain reaction

Quantitative real-time polymerase chain reaction (qRT-PCR) was used to assess mRNA expression. Taqman® PCR master mix and fluorescently tagged Taqman® primers (Supplemental Table 1) (Primer design, Southampton, UK) were used. Fluorescence was measured using the ViiA7™ real-time PCR System (Life Technologies, Paisley, UK). The cycle conditions were 50℃ for 2 min, 95℃ for 10 min and 40 cycles of 95℃ for 15 s, 60℃ for 1 min.

### Cx43 siRNA

siRNA duplex sequences targeted to Cx43 (TriFECTa®RNAi Kit from Integrated DNA Technologies, Tyne and Wear, UK) were used to knockdown Cx43 gene expression. A scrambled siRNA was used as a control. siRNA transfection was carried out using Lipofectamine 3000 transfection reagent (Invitrogen, Paisley, UK). Lipofectamine 3000 was mixed with the siRNAs and incubated for 15 min at room temperature allowing complexes to form. rPAFs were then transfected with a final concentration of 5 nM siRNA. Proliferation and migration assays were performed 24 h post transfection. Transfection of rPAFs with siRNA targeting Cx43 resulted in approximately 50% reduction in Cx43 protein expression (Supplemental Fig. 1).

**Fig. 1.**
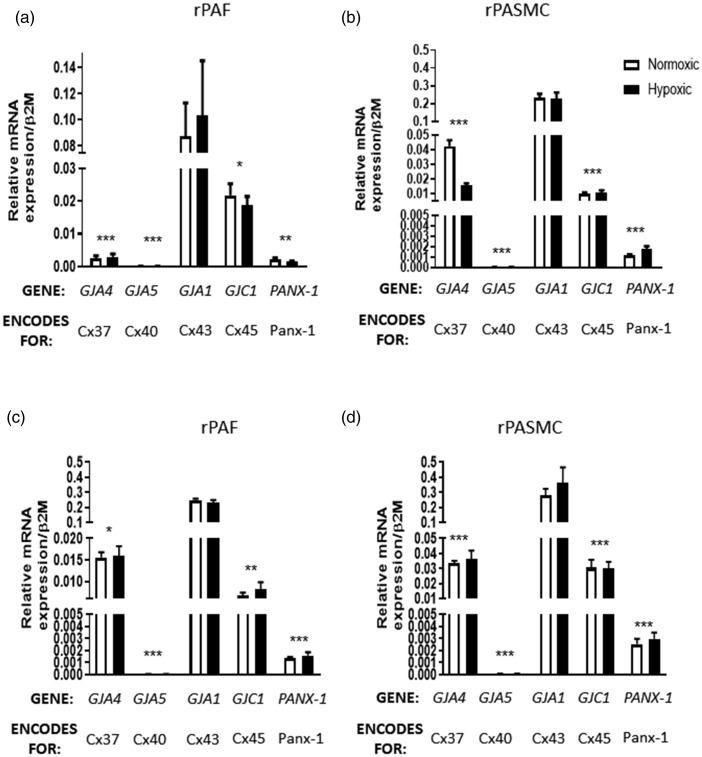
Expression of connexins in rPAFs (a) and rPASMCs (b) in serum-free media and in rPAFs (c) and rPASMCs (d) in media containing 1% serum. In both cell types, under serum-free conditions and in the presence of 1% serum, *GJA1* was found to be the most predominant connexin gene expressed, while *GJA5* was expressed although at very low levels. In hypoxic conditions, no changes in expression of *GJA4*, *GJA5*, *GJA1*, *GJC1* or *PANX-1* were found in rPAFs or in rPASMCs. Data expressed as mean ± SEM, and analysis was carried out by two-way ANOVA with a Dunnett’s multiple comparison test. *p < 0.05, **p < 0.01, ***p < 0.001, vs. *GJA1*, *n* = 6. rPAF: rat pulmonary artery fibroblasts; rPASMC: rat pulmonary artery smooth muscle cells.

### Proliferation assays

To determine proliferation, confluent 75 cm^2^ flasks of rPAFs or rPASMCs were split, and 1/12 of the cells were evenly distributed over 2 × 24 well plates. Cells were grown in 10% FBS DMEM to 50% to 60% confluency and then quiesced in SFM for 24 h. After being quiesced, 0.1% FBS, 1% FBS or 1% FBS in the presence of Gap27 (300 µM) or Cx43 siRNA (5 nM) was added before cells were placed in normoxia or hypoxia for 24 h. 1% FBS was used as it has previously been shown to promote PAF proliferation in hypoxia.^17,24^ We wished to verify that 0.1% FBS did not cause proliferation of cells in the presence of hypoxia, and therefore, 0.1% FBS could be used for subsequent migration assays. Cells were counted using an automated cell counter (Countess II, Life Technologies, UK). Cell culture media was aspirated, and cells were washed in 500 μl of sterile PBS. After removing the PBS, 100 μl of trypsin (Life Technologies, UK) was added to each well. The plate was placed at 37℃ to aid the detachment of the cells. The detached cells were re-suspended in 700 μl of SFM per well and mixed. The suspended cells were centrifuged at 2600 × g for 5 min, and the pellet was then re-suspended in equal volumes of SFM and trypan blue and loaded onto glass slides and inserted into the Countess II to determine cell concentration per ml.

### Migration assays

Cells were seeded into 6-well plates and left to grow until 100% confluent. Once fully confluent, the cells were quiesced in SFM for 24 h, and an initial scratch was made in each well and was photographed. After being quiesced, 0.1% FBS or 0.1% FBS in the presence of Gap27 (300 µM) or Cx43 siRNA (5 nM) was added to the cells for 24 h normoxic or hypoxic exposure. After 24 h, the scratch was photographed for comparison with the previous images. Cell migration was analysed by measuring the changes in scratch width and converting this into a percentage migration.

### Statistics

Data were analysed using a two-way analysis of variance (ANOVA) followed by either a Tukey post hoc test or Dunnett’s multiple comparison test as appropriate. A value of *p* < 0.05 indicated statistical significance.

## Results

### Effects of hypoxia on connexin gene expression in rPAFs and rPASMCs

The presence of mRNA for *GJA4* (encoding Cx37*)*, *GJA5* (encoding Cx40), *GJA1* (encoding Cx43), *GJC1* (encoding Cx45) and *PANX-1* in rPASMCs and rPAFs in SFM (Fig. 1a and b) and 1% serum conditions (Fig. 1c and d) was confirmed using qRT-PCR. In both cell types, *GJA1* was found to be the most predominant connexin gene expressed, while *GJA5* was expressed only at very low levels. After 24 h of hypoxia, no changes in expression of *GJA4*, *GJA5*, *GJA1*, *GJC1* or *PANX-1* were found in rPAFs (Fig. 1a and c) or rPASMCs (Fig. 1b and d). *GJA1* expression was upregulated in rPAFs exposed to 1% serum conditions compared to those in SFM, while there was no change in expression of *GJA4*, *GJA5*, *GJC1* or *PANX-1* between rPAFs cultured in SFM or 1% serum (Supplemental Fig. 2). There was no change in expression of *GJA1*, *GJA4*, *GJA5*, *GJC1* or *PANX-1* between rPASMCs cultured in SFM or 1% serum (Supplemental Fig. 2).

**Fig. 2.**
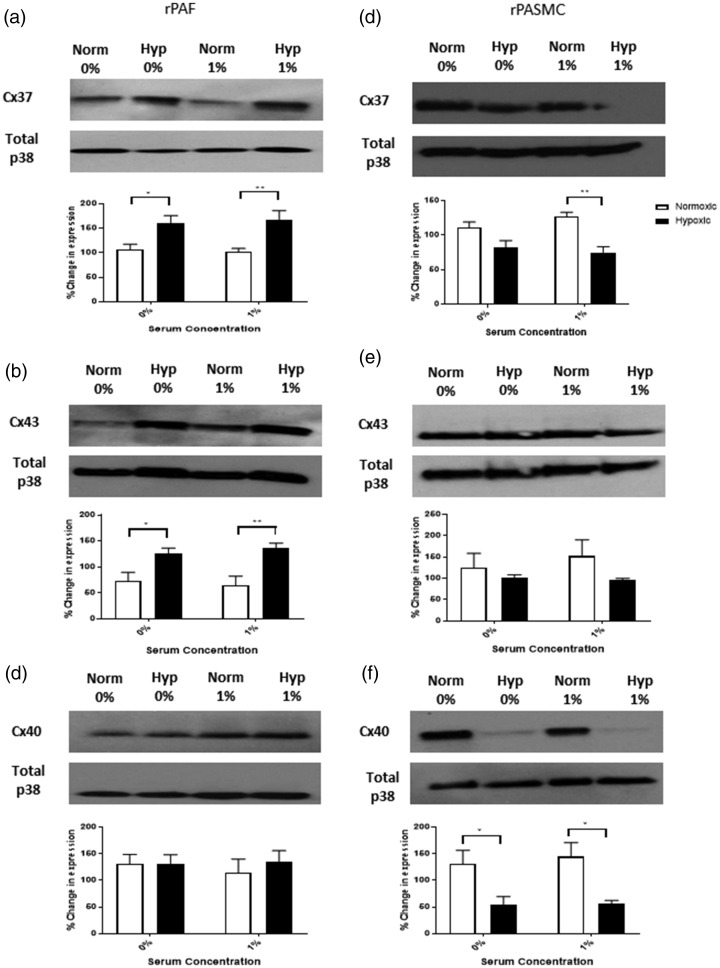
Protein expression of Cx37, Cx40 and Cx43 in rPAFs and rPASMCs. In rPAFs, hypoxia increased Cx37 (a) and Cx43 (b) protein expression, while Cx40 expression was unchanged (c). In rPASMCs, hypoxia decreased Cx37 expression under 1% serum conditions (d). Cx43 expression was unchanged by hypoxia in rPASMCs (e), and decreased Cx40 expression was observed under both serum free and 1% serum conditions (f). Data expressed as mean ± SEM, and analysis was carried out by two-way ANOVA with a Tukey post hoc test. *p < 0.05, **p < 0.01, *n* = 4–5. rPAF: rat pulmonary artery fibroblasts; rPASMC: rat pulmonary artery smooth muscle cells; Cx37: connexin 37; Cx43: connexin 43; Cx40: connexin 40.

### Effects of hypoxia on connexin protein expression in rPAFs and rPASMCs

Connexin protein expression was examined using Western blot analysis. We focused on Cx37, Cx40 and Cx43 as they have previously been shown to have a role in regulation of the pulmonary vasculature.^10,11,15^ In rPAFs in both SFM and 1% FBS, hypoxic exposure increased protein expression of Cx37 (Fig. 2a) and Cx43 (Fig. 2b) but had no effect on protein expression of Cx40 (Fig. 2c). In rPASMCs, hypoxic exposure in the presence of 1% FBS decreased protein expression of Cx37, with no effect observed in the presence of SFM (Fig. 2d). In both SFM and 1% FBS, hypoxic exposure had no effect on protein expression of Cx43 (Fig. 2e) and decreased protein expression of Cx40 (Fig. 2f).

### Cx43 inhibition prevents cell proliferation in rPAFs but not rPASMCs

**Fig. 3.**
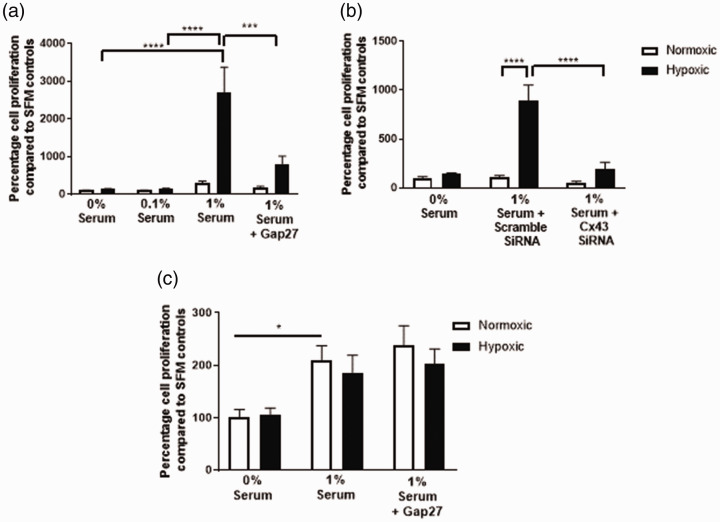
Effects of Cx43 inhibition on hypoxia-induced proliferation of rPAFs and rPASMCs. In rPAFs, a significant increase in cell proliferation was observed in hypoxic cells. Gap27 (a) and Cx43 siRNA (b) were both shown to decrease proliferation in hypoxic rPAFs. Hypoxia did not alter rPASMC proliferation (c). Data are expressed as mean ± SEM, and analysis was carried out by two-way ANOVA with a Tukey post hoc test. *p < 0.05, ***p < 0.001, ****p < 0.0001, *n* = 8–9. rPAF: rat pulmonary artery fibroblasts; rPASMC: rat pulmonary artery smooth muscle cells; SFM: serum-free media.

Hypoxic exposure resulted in increased proliferation of rPAFs only in the presence of 1% FBS. Hypoxic exposure did not result in proliferation of rPAFs cultured in SFM or 0.1% FBS (Fig. 3a and b). Proliferation of rPAFs in response to hypoxia in the presence of 1% FBS was inhibited by Gap27 (Fig. 3a) or Cx43 siRNA (Fig. 3b). rPAFs did not proliferate when cultured in SFM, 0.1% or 1% FBS under normoxic conditions, and the addition of Gap27 or Cx43 siRNA had no further effect (Fig. 3a and b). rPASMCs proliferated in response to 1% FBS under normoxic conditions. Hypoxia had no further effects on FBS-induced proliferation in rPASMCs. Gap27 did not inhibit FBS-induced proliferation of rPASMCs (Fig. 3c). Cell proliferation images are shown in Supplemental Fig. 3.

### Cx43 inhibition prevents cell migration in rPAFs but not rPASMCs

**Fig. 4.**
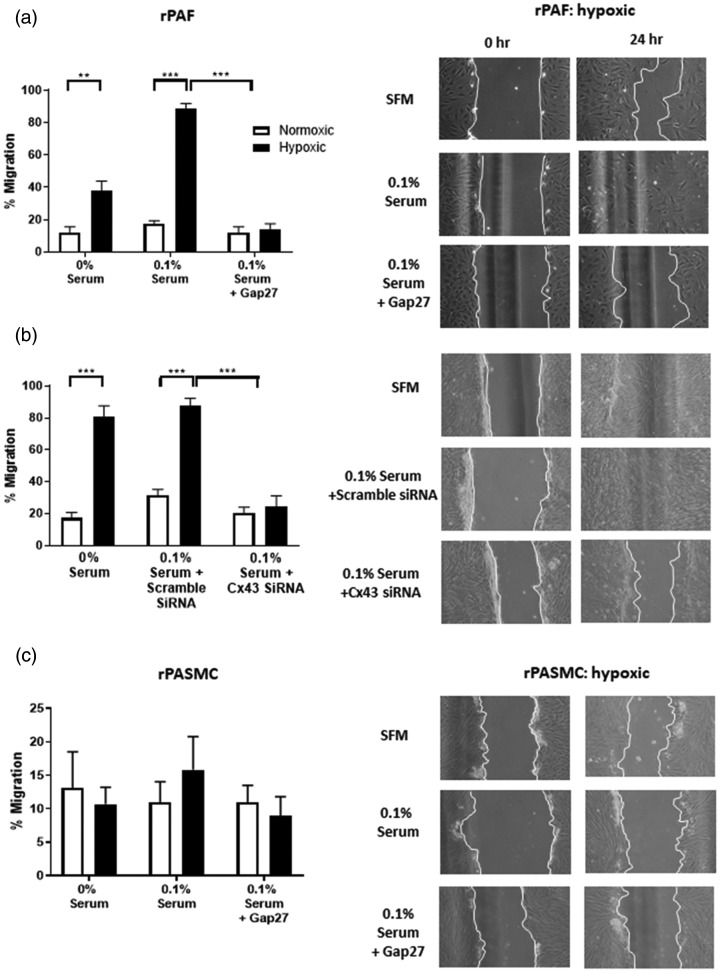
Effects of Cx43 inhibition on hypoxic-induced migration of rPAFs and rPASMCs. A significant increase in migration was observed in rPAFs in response to hypoxia. This hypoxia-mediated increase was prevented with the addition of Gap27 (a, upper panel) or Cx43 siRNA (b, central panel). Hypoxia did not alter rPASMC migration which was also unaltered with the addition of Gap27 (c, lower panel). The column on the right shows an example of the scratch assay, while quantitative data from 4 to 9 assays are shown in the column on the left. Images are 10× magnification. Data expressed as mean ± SEM, and analysis was carried out by two-way ANOVA with a Tukey post hoc test. **p < 0.01, ***p < 0.001. rPAF: rat pulmonary artery fibroblasts; rPASMC: rat pulmonary artery smooth muscle cells; SFM: serum-free media.

Having established that rPAFs cultured in hypoxic conditions in the presence of 0.1% FBS do not show a proliferative response (Fig. 3a), we used this serum concentration to study the effects of hypoxia on cellular migration. Hypoxia resulted in an increase in rPAF migration, which was inhibited by either Gap27 (Fig. 4a) or Cx43 siRNA (Fig. 4b). In rPASMCs, hypoxia did not alter the rate of migration, and Gap27 had no effects on the rate of migration (Fig. 4c). Neither rPAFs nor rPASMCs migrated under normoxic conditions (Supplemental Fig. 4).

### Gap27 inhibits phosphorylation of p38 MAPK and ERK MAP kinase activity in rPAFs exposed to acute hypoxia

**Fig. 5.**
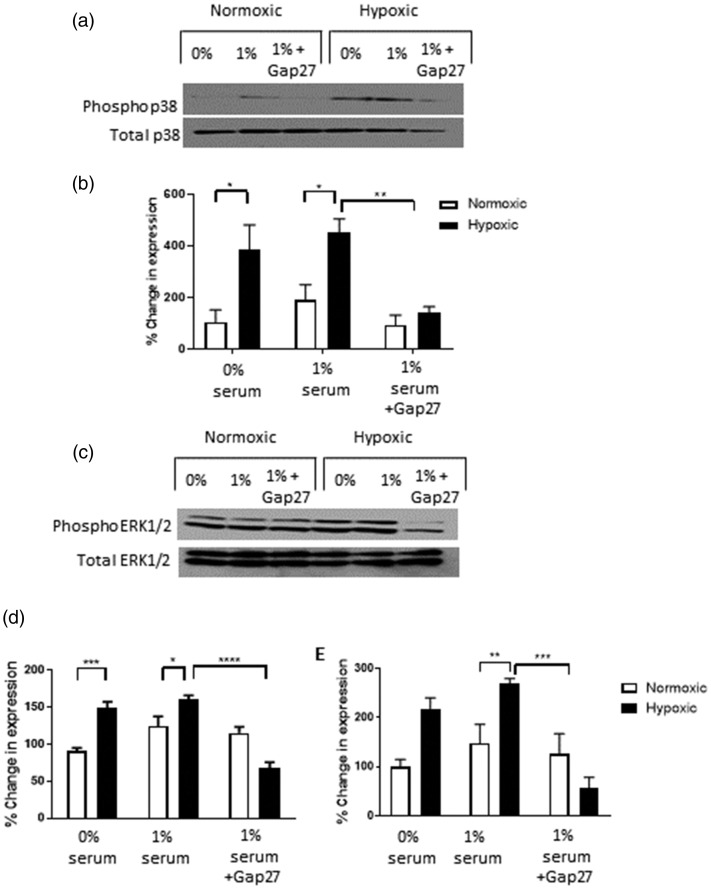
Effects of Gap27 on MAPK expression in rPAFs. Hypoxia increased phosphorylated p38 (a and b) and phosphorylated ERK1/2 (c–e) expression in rPAFs, an effect prevented by Gap27. Western blot of phosphorylated p38 shown in (a) and quantitative analysis in (b). While Western blot of phosphorylated ERK1/2 is shown in (c) and quantitative analysis of ERK1 in (d) and ERK2 in (e). Data expressed as mean ± SEM, and analysis was carried out by two-way ANOVA with a Tukey post hoc test. Lanes 1 and 4 contain 0% serum, Lanes 2 and 5 contain 1% serum and Lanes 3 and 6 contain 1% serum + 300 µM of Gap27. *p < 0.05, **p < 0.01, ***p < 0.001, ****p < 0.0001, *n* = 4.

Phosphorylation of both p38 MAPK and EE1/2 MAPK has previously been shown to play a role in hypoxic-induced proliferation and migration of rPAFs.^2^ As we have shown Gap27 to inhibit hypoxic-induced rPAF proliferation and migration, we investigated if Gap27 altered the phosphorylation of p38 MAPK and ERK1/2 MAPK. In the present study, hypoxia caused an increase in phosphorylation of both p38 MAPK and ERK1/2 MAPK, both of which were inhibited in the presence of Gap27 (Fig. 5a and b).

### Connexin 43 expression in whole lung tissue from sugen5416/hypoxic rats treated with an ASK1 inhibitor

**Fig. 6.**
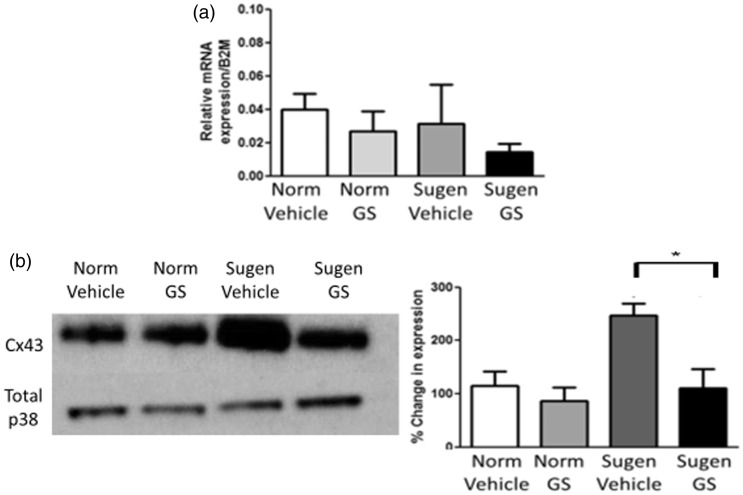
Connexin 43 gene and protein expression in sugen5416/hypoxic lung tissue. Sugen5416/hypoxic rats showed no changes in *GJA1* (Cx43) gene expression compared to control rats. The ASK-1 inhibitor GS-444217 also had no effect on gene expression of *GJA1*, *n* = 5–7 (a). Increased Cx43 protein expression was observed in sugen5416/hypoxic rats in comparison to control rats. This increase in Cx43 protein expression was reversed in sugen5416/hypoxic rats treated with the ASK-1 inhibitor GS-444217 (*n* = 4), Western blot shown in (b) and quantitative analysis in (c). Data expressed as mean ± SEM, and analysis was carried out by two-way ANOVA with a Tukey post hoc test. *p < 0.05. Cx43: connexin 43.

To complement the changes observed in connexin expression in our cellular model of PH, we investigated changes in Cx43 gene and protein expression ex vivo in whole lung tissue derived from sugen5416/hypoxic rats. Gene expression of Cx43 remains unchanged (Fig. 6a); however, protein expression of Cx43 was increased in whole lung tissue from sugen5416/hypoxic rats (Fig. 6b and c). Interestingly, sugen5416/hypoxic rats dosed with the ASK-1 inhibitor GS-444217, which attenuated PH in these rats,^17^ did not show an increase in Cx43 protein expression.

## Discussion

To our knowledge, this is the first study to investigate the role of Cx43 in rPAFs in response to hypoxia. The data presented here show that in rPAFs, Cx43 protein expression is increased by hypoxia and that Cx43 plays a role in hypoxic-induced proliferation and migration as well as hypoxic-induced phosphorylation of both p38 and ERK1/2 MAPK. Cx43 protein expression is also upregulated in whole lung tissue derived from sugen5416/hypoxic rats, an effect dependent upon the ASK-1/MAPK pathway.

Interestingly, a previous study showed that Cx43 expression was increased in pulmonary arteries from patients with chronic hypoxic PH and that mice heterozygous for Cx43 were protected against hypoxic-induced pulmonary vascular remodelling.^9^ Thus, the data presented in this manuscript build on the hypothesis that Cx43 may be a promising therapeutic target for the treatment of PH associated with chronic hypoxia.

In this study, it was confirmed that *GJA1*, which encodes Cx43, is the predominantly expressed connexin gene in both rPAFs and rPASMCs. This is similar to many other cell types such as keratinocytes, bone cells and fibroblast skin cells where *GJA1* is the predominant connexin gene expressed.^25,26^ Although mRNA expression of the majority of connexins studied remained unaltered following 24 h hypoxic exposure in both rPAFs and rPASMCs, *GJA4*, which encodes Cx37, was downregulated in rPASMCs. This is in line with other studies which found that *GJA4* gene expression was reduced in the right ventricular tissue of pulmonary artery banded mice^27^ and also in PAECs from PAH patients.^14^ In the current study, hypoxia downregulated protein expression of Cx37 and Cx40 in rPASMCs and increased protein expression of Cx37 and Cx43 in rPAFs. In PAH patients, a recent study showed that both Cx37 and Cx40 mRNA and protein expression were decreased in isolated PAECs and in lung endothelial layers.^28^ The rat monocrotaline model also showed decreased Cx40 gene and protein expression in both whole lung and isolated PASMC,^12^ and the mouse hypoxic model showed decreased Cx40 gene expression in the pulmonary artery.^8^ The increased Cx43 protein expression observed in the current study in response to hypoxia aligns with previous studies which have shown Cx43 expression to be increased in pulmonary arteries from patients with chronic hypoxic PH and also in pulmonary arteries from chronic hypoxic rats as well as monocrotaline rats.^9,28,29^ Conversely, using a C57BL6 mouse model subjected to 14 days of hypoxia, we showed a decrease in Cx43 gene and protein expression in the pulmonary artery,^11^ while another study found a 21-day hypoxic exposure to have no effect on CX43 gene expression in the CD1 mouse pulmonary artery.^9^ Thus, Cx43 may be differentially regulated by hypoxia depending upon the strain/species as well as the duration of hypoxic exposure. While we may expect a correlation between changes in gene expression and changes in protein expression, this correlation can be as low as 40% in eukaryotes. Post-transcriptional regulation and rates of production and degradation of mRNA and protein molecules play a role in variation between mRNA and protein abundance.30 For example, in mammalian cells, the rate of production of mRNA is generally much lower than that of production of protein.^31^

Increased proliferation and migration of rPAFs in response to hypoxia is well established and is thought to be important in hypoxic-induced remodelling of the pulmonary vasculature.^2^ We showed Gap27 or siRNA directed against Cx43 to inhibit the hypoxic-induced proliferative and hypoxic-induced migratory responses observed in rPAFs, thus providing evidence that Cx43 plays a role in these responses. As we did not determine expression of other connexins in rPAFs in response to Gap27 or siRNA directed against Cx43, we cannot rule out that other connexins may also be involved in these responses. Until now, studies of connexins in the vasculature have focused on endothelial and smooth muscle cells, with little known about connexins in vascular fibroblasts. However, the role of connexins in migration has been studied in skin fibroblasts. Contrary to our findings, Gap27 and siRNA directed against Cx43 have been shown to increase the rates of migration in primary juvenile human dermal fibroblasts.^32–34^ Interestingly, however, Gap27 did not affect migration in human neonatal or adult dermal fibroblasts.^34^ These disparities could be due to species variation or differences in cell types. In the current study, proliferation of rPAFs was determined using an automated cell counter which gives living and dead cell concentrations; therefore, we could rule out Gap27 or Cx43 siRNA causing cell death. Hypoxic-induced migration of rPAFs was observed using low serum concentrations (0.1% FBS) which were not sufficient to uncover the hypoxic-induced proliferative response, therefore confirming that migration was independent of proliferation. In line with our results, connexins, and in particular Cx43, have been shown to play a role in cancer cell migration and proliferation.^35,36^ In rat hepatocellular carcinoma cells, silencing Cx43 with siRNA resulted in a 30% decrease in cell migration, whereas in bone cells, increases in Cx43 showed a three- to fourfold increase in cell proliferation.^37^ In the current study, rPASMCs proliferated in response to 1% FBS to a similar degree under both normoxic and hypoxic conditions. While our finding that rPASMCs do not proliferate in response to hypoxia is in agreement with some previous studies,^17,38^ others have shown hypoxic-induced proliferation of rPASMCs. You et al. reported that rPASMCs proliferated when exposed to hypoxic conditions of 3% O_2_ for 48 h (5% O_2_ for 24 h was used in the current study). It is of interest that Jiang et al. exposed rPASMCs to 3% O_2_ for 24, 48 and 72 h, observing only a small increase in proliferation at 24 h, with the largest increase in proliferation observed at 72 h.^39,40^ In the current study, rPASMC and rPAF proliferation was assessed under identical experimental conditions. It is of interest to note that the rPASMCs studied are capable of proliferation as this was observed in response to serum.

As expected, FBS-induced proliferation of rPASMCs and our study found Gap27 to have no effect on this. Previous studies have shown connexins to mediate systemic vascular SMC proliferation and migration. MAPK phosphorylation of Cx43 has been shown to be critical in PDGF-induced proliferation of mouse aortic smooth muscle cells.^16^ In saphenous vein SMCs, an over-expression of Cx43 caused an increase in cellular proliferation and migration in response to angiotensin II, whereas Cx43 siRNA inhibited these effects.^41^ A possible explanation for the disparity between these results and those of the current study could be due to differences between the pulmonary and systemic circulation.^42^ Different responses between the pulmonary and systemic circulation have been well documented with one example being chronic hypoxia resulting in increased cell proliferation via p38 MAPK in PAFs but causing no change in aortic fibroblasts or mesenteric fibroblasts.^43^

Having established that inhibition of Cx43 caused changes in rPAF function, we wished to examine the signalling mechanism involved in these changes. Previous research has shown that the MAPK pathway, and in particular p38 MAPK, is key in hypoxic-induced proliferation and migration of rPAFs.^44^ It has also been shown that phosphorylation of ERK1/2 is increased by hypoxia in rPAFs.^44^ In the current study, Gap27 inhibited phosphorylation of p38 MAPK and ERK1/2 in hypoxic rPAFs. This suggests that the MAPK-mediated proliferation and migration which occurs in rPAFs requires connexin-mediated signalling. Evidence from the literature suggests reciprocal interactions between these pathways rather than one pathway being upstream of the other. For example, inhibition of Cx43 has been shown to lead to a decrease in phosphorylation of ERK1/2 and decreased cellular proliferation, while pharmacological inhibition of ERK1/2 reduced expression of Cx43 in human umbilical vein endothelial cells.^45^ In addition, Cx43 has MAPK phosphorylation sites located on the carboxyl tail and has previously been shown to augment p38 MAPK-mediated cell migration.^15^ MAPK phosphorylated Cx43 has also been shown to interact with cyclin E in mouse vascular SMCs, and this interaction is critical for vascular SMC proliferation.^16^ p38MAPK/ERK signalling has been shown to induce membrane Cx43 internalisation in osteocyte-like MLO-Y4 cells.^46^ On the other hand, Cx43 dephosphorylation at serine 282 activates the p38 pathway in cardiomyocytes,^47^ and opening of Cx43 hemichannels has been shown to activate p38 MAPK in mouse cortical astrocytes.^48^ In future studies, it would be of interest to further investigate the relationship between Cx43 and the MAPK pathway in rPAFs.

ASK-1 is a member of the MAPK family that becomes auto-phosphorylated in response to oxidative stress. Once phosphorylated, ASK-1 activates other downstream members of the MAPK pathway such as p38 MAPK and c-Jun N-terminal kinase.^49^ ASK-1 inhibition decreases rPAF migration and proliferation in response to 24 h hypoxia.^17^ In the sugen5416/hypoxic rat model of PH, inhibiting ASK-1 decreased right ventricular pressure and vascular remodelling *in vivo*.^17^ *In vitro* rPAFs derived from sugen5416/hypoxic rats displayed increased proliferation, migration and p38 MAPK expression; these effects were not present in rPAFs derived from sugen5416/hypoxic rats dosed with the ASK1 inhibitor, GS-444217.^17^ Using lung tissue from these sugen5416/hypoxic rats, we investigated if the changes seen *in vivo* and *in vitro* also coincided with an altered connexin expression. We observed no changes in *GJA1* (Cx43) gene expression in lung tissue from sugen5416/hypoxic rats. Cx43 protein expression was increased in the lung tissue from these rats; however, this increased Cx43 protein expression was not observed in the lung tissue of rats treated with GS-444217. These observations provide further evidence for interactions between the MAPK and Cx43 signalling pathways.

Interestingly, the results in whole lung tissue display a similar pattern to that observed in rPAFs, with hypoxia altering protein expression but not gene expression of Cx43. As noted earlier, changes in gene and protein expression frequently do not correlate.^30^ Previous studies have also found a possible link between Cx43 and ASK-1. Interactions between Cx43 and ASK-1 were identified via co-immunoprecipitation experiments in primary astrocytes,^50^ and an interaction between Cx43 and ASK1 is thought to mediate apoptosis in human embryonic kidney cells.^51^

In conclusion, we have shown that in rPAFs, Cx43 has an important role in mediating hypoxic-induced proliferation and migration, via increased phosphorylation of p38 MAPK and ERK1/2. We have also shown that Cx43 protein expression is increased in lung tissue from the sugen5416/hypoxic model. Thus, the present study provides further evidence that Cx43 is involved in aberrant cellular proliferation associated with the remodelling of the pulmonary vasculature in response to hypoxia, the precise mechanism of which warrant further investigation.

## Supplemental Material

sj-pdf-1-pul-10.1177_2045894020937134 - Supplemental material for Connexin 43 plays a role in proliferation and migration of pulmonary arterial fibroblasts in response to hypoxia Click here for additional data file. Supplemental material, sj-pdf-1-pul-10.1177_2045894020937134 for Connexin 43 plays a role in proliferation and migration of pulmonary arterial fibroblasts in response to hypoxia by Andrew J. McNair, Kathryn S. Wilson, Patricia E. Martin, David J. Welsh and Yvonne Dempsie in Pulmonary Circulation
