# Supplementary material for: Connexin 43 plays a role in proliferation and migration of pulmonary arterial fibroblasts in response to hypoxia
Source: Pulm Circ. 2020 Jul 6;10(3):2045894020937134. doi: 10.1177/2045894020937134 (PMC7338651; doi:10.1177/2045894020937134)
Supplement: sj-pdf-1-pul-10.1177_2045894020937134 - Supplemental material for Connexin 43 plays a role in proliferation and migration of pulmonary arterial fibroblasts in response to hypoxia [file sj-pdf-1-pul-10.1177_2045894020937134.pdf]

<u>Connexin 43 plays a role in the remodelling processes observed in both *in vitro* and *in vivo* experimental model of pulmonary hypertension</u>

<u>Supplementary Figures</u>

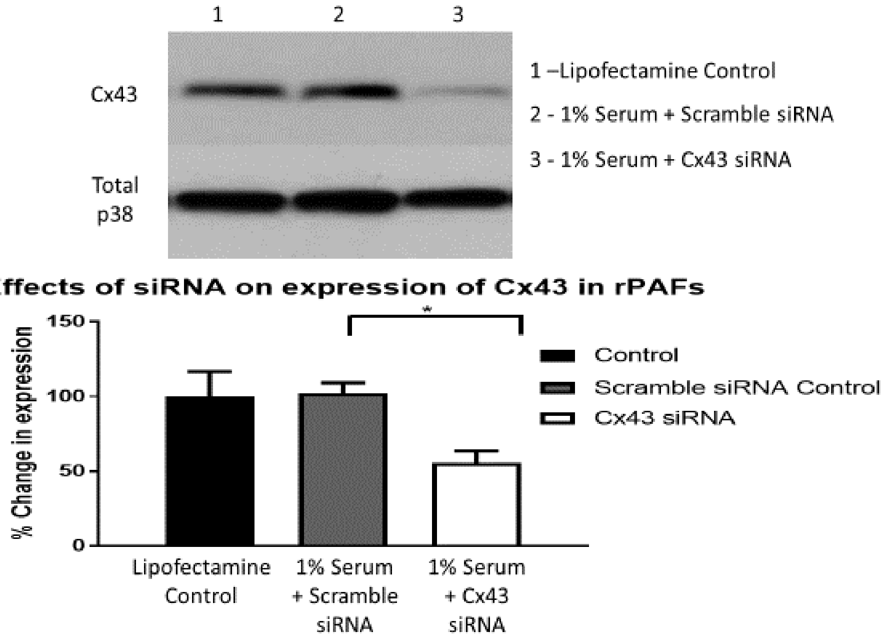

Supplemental figure 1 Effects of Cx43 siRNA on protein expression of Cx43 in rPAFs. Cx43 siRNA caused a significant decrease in Cx43 protein expression compared to scrambled siRNA and Lipofectamine only controls. Data expressed as mean ± sem and were analysed one way ANOVA followed by Bonferroni's post-hoc test. n =4

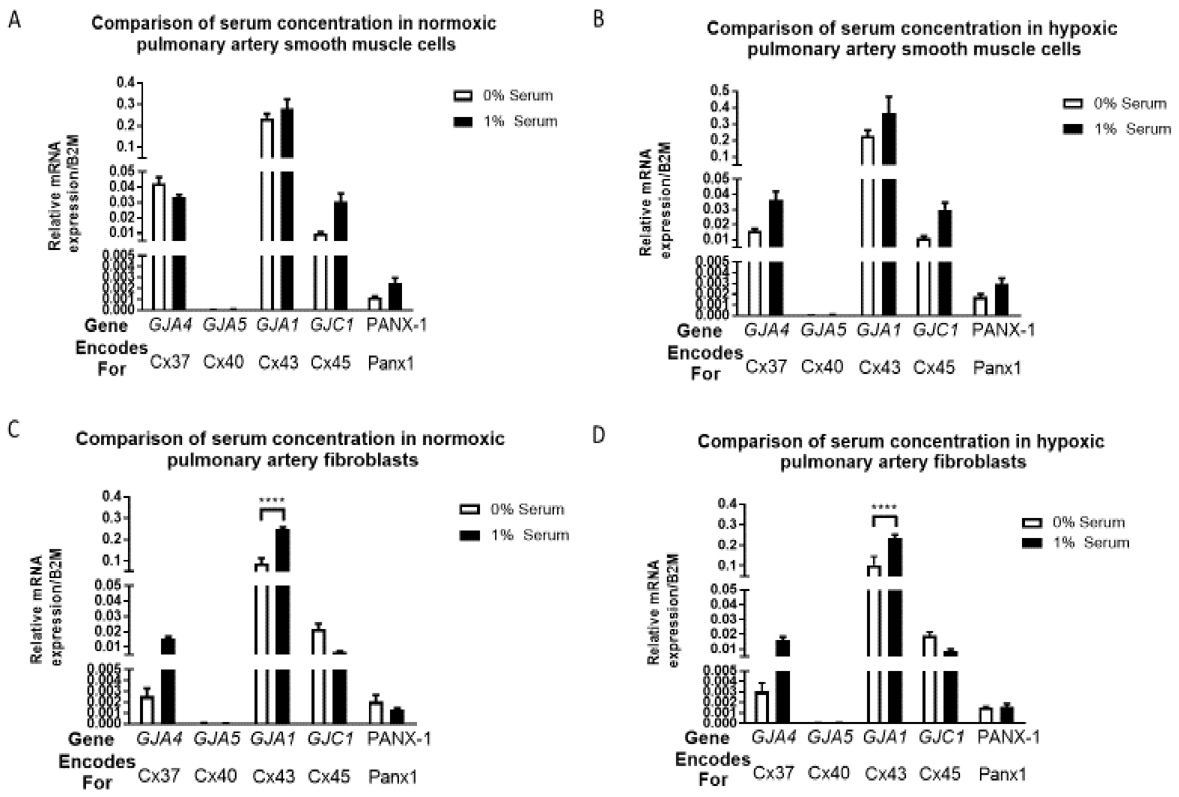

Supplemental figure 2. Effects of serum on connexin and pannexin gene expression in normoxic and hypoxic rPASMCs and rPAFs. Serum had no effect on expression of the genes *GJA4, GJA5, GJA1, GC1* or *PANX-1* in rPASMCs under either normoxic or hypoxic conditions. *GJA1* expression was increased by serum in rPAFs under both normoxic and hypoxic conditions. Expression of *GJA4, GJA5, GC1* or *PANX-1* were unaffected by serum in rPAFs under either normoxic or hypoxic conditions. Data expressed as mean ± sem and were analysed one way ANOVA followed by Bonferroni's post-hoc test. n =6

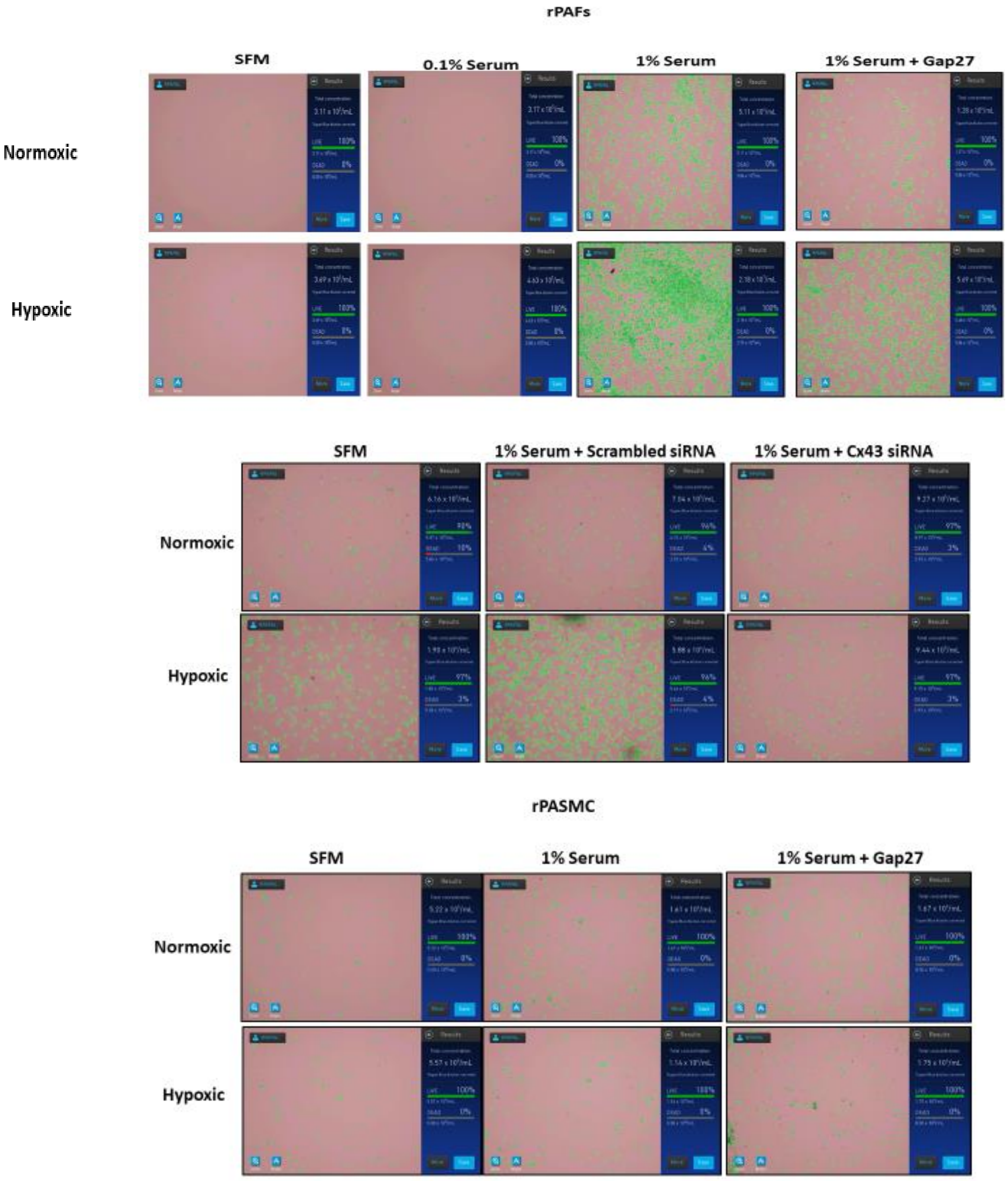

Supplemental figure 3 Cell Proliferation images captured by the automated cell counter Countess II (Life Technologies, United Kingdom). Hypoxia increased cell proliferation in rPAFs (A and B) but not rPASMCs (C). Hypoxic-induced proliferation of rPAFs was inhibited by Gap27 (A) and Cx43 siRNA (B). n=8-12

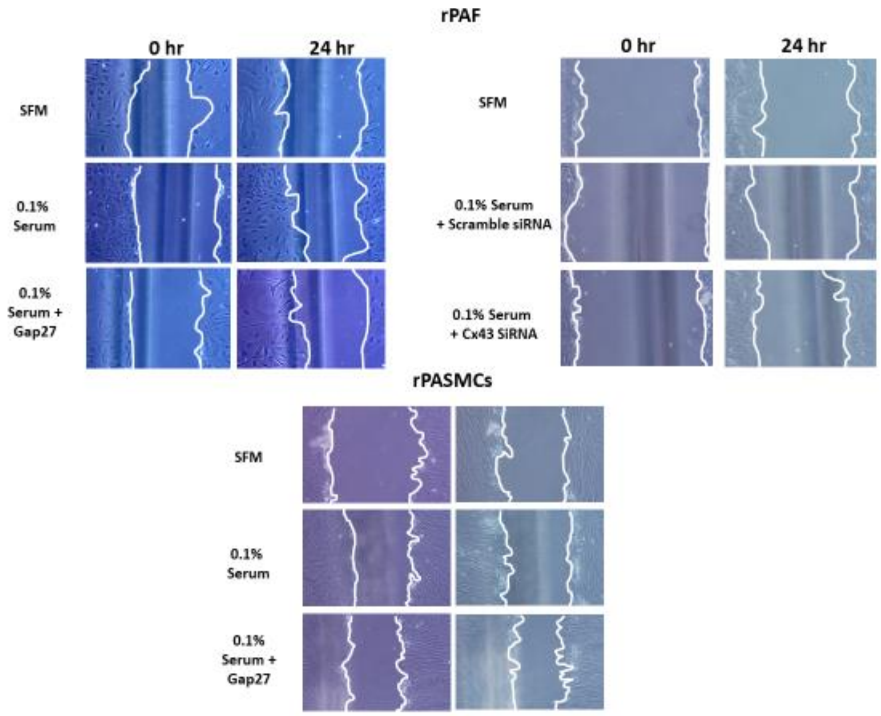

Supplemental figure 4. Effects of Cx43 inhibition on migration of rPAFs and rPASMCs under normoxic conditions. There were no changes in migration of rPAFs or rPASMCs under normoxic conditions. Data expressed as mean ± SEM followed two-way ANOVA followed by tukey's post-hoc test. n=4-6

Supplementary Methods

RNA Analysis

Protocol described in full in the main manuscript. The primer sequences used for the qRT-PCR are shown in the table below

**Primer Name**	**Sequence**
Rat Connexin 37	Forward Primer - CCCATCTCCCACATCCGATAC<br>Reverse Primer - TCCCTCTTTCTGCCGCAAC
Rat Connexin 40	Forward Primer - GCAGGACAGAAGCGGTCTC<br>Reverse Primer - GATTAATGAGCAACACCATCCAGA
Rat Connexin 43	Forward Primer - GAAGCACCATCTCCAACTCG<br>Reverse Primer - TTCATGTCCAGCAGCAACTTT
Rat Connexin 45	Forward Primer - ACCAATTCCACCACCATGAGT<br>Reverse Primer - TCAGCACAGTGAGCCAGATC
Rat Pannexin 1	Forward Primer - ACCTCTGCTCCGACCTGAA<br>Reverse Primer - CACATTCTCAGAGTCACTCCTGGA

Table 1 – Primer sequences for qPCR
